# The role of thalamic group II mGlu receptors in health and disease

**DOI:** 10.1042/NS20210058

**Published:** 2022-11-15

**Authors:** Caroline S. Copeland, Thomas E. Salt

**Affiliations:** 1 Centre for Pharmaceutical Medicine, Institute of Pharmaceutical Sciences, Kings’ College London, U.K.; 2 Institute of Ophthalmology, University College London, U.K.; 3 Neurexpert Ltd., Newcastle, U.K.

**Keywords:** astrocytes, glutamate receptor, synapses, thalamus

## Abstract

The thalamus plays a pivotal role in the integration and processing of sensory, motor, and cognitive information. It is therefore important to understand how the thalamus operates in states of both health and disease. In the present review, we discuss the function of the Group II metabotropic glutamate (mGlu) receptors within thalamic circuitry, and how they may represent therapeutic targets in treating disease states associated with thalamic dysfunction.

## Introduction

**Figure 1:**
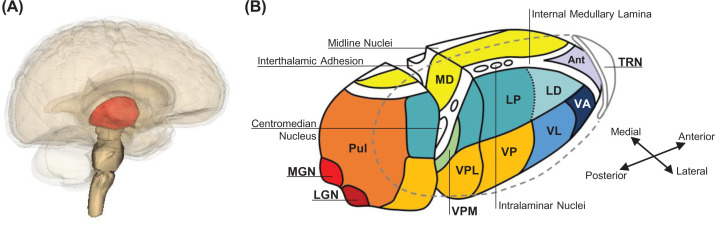
Overview of Thalamic Nuclei (**A**) Location of the thalamus within the human brain. (**B**) Major nuclei comprising the thalamus. The thalamus in (**A**; red) has been enlarged in (**B**) to enable inspection of the major thalamic nuclei. Abbreviations: Ant, anterior; LD, dorsal lateral nucleus; LGN, lateral geniculate nucleus; LP, lateral posterior nucleus; MD, mediodorsal nucleus; MGN, medial geniculate nucleus; Pul, pulvinar; TRN, thalamic reticular nucleus; VA, ventral anterior nucleus; VL, ventral lateral nucleus; VP, ventral posterior nucleus; VPL, ventral posterolateral nucleus; VPM, ventral posteromedial nucleus. (**A**) reproduced under Creative Commons License CC BY-SA 2.1 JP.

The anatomically central position of the thalamus within the brain (Figure 1A) is functionally appropriate given its central role in the processing of almost all sensory, motor, and cognitive information: information entering the brain is first channelled to the relevant thalamic nuclei for processing and integration with other inputs prior to its subsequent dispatch to the corresponding cortical area(s). Consequentially, thalamic processing underpins the multimodal and multilevel functioning of the central nervous system (CNS) making it critical to understand how the thalamus operates. In recent years, there has been considerable work to understand how the Group II metabotropic glutamate (mGlu) receptors contribute to thalamic processing. Maintenance of the original spatiotemporal quality of stimuli through inhibition of top-down modulatory processes in the thalamus appears to be mediated by the Group II mGlu receptors, which have made them potential targets for therapeutic intervention to treat schizophrenia, pain, and epilepsy. In the present review, we describe the fundamentals of thalamic circuitry and Group II mGlu receptor pharmacology, how they interact, and their potential roles in health and disease.

## A brief overview of thalamic circuitry

The thalamus comprises over 30 distinct nuclei (Figure 1B) [1], with each nucleus governing the processing and relay of information to a discrete area of corresponding cerebral cortex. Information across all modalities, with the possible exception of olfaction, is relayed by thalamic neurons (sensory, motor, cognitive) [2,3]. These thalamic relay neurons receive information in two forms, namely driver inputs and modulatory inputs, which can be distinguished by their terminal morphology and synaptic location [3]: driver inputs have characteristic large round terminals that form synapses proximal to the soma of thalamic relay neurons, whereas modulatory inputs have small round terminals that form synapses distally onto the dendritic trees of thalamic relay neurons [3–6]. The source of information transmitted by driver inputs determines the classification of the thalamic nuclei they innervate as either first-order or higher-order nuclei, with first-order nuclei receiving and processing driver inputs from the periphery (e.g., visual, auditory, somatosensory), and higher-order nuclei receiving and processing driver inputs from cortical layer V [3–6] (note: some thalamic nuclei receive driver inputs from both the periphery and the cortex – for example, the posterior medial thalamus receives somatosensory driver inputs from both the periphery and cortical driver inputs from somatosensory, motor, perirhinal, and insular cortices [7–9] – such nuclei are considered mixed nuclei [10]).

**Figure 2:**
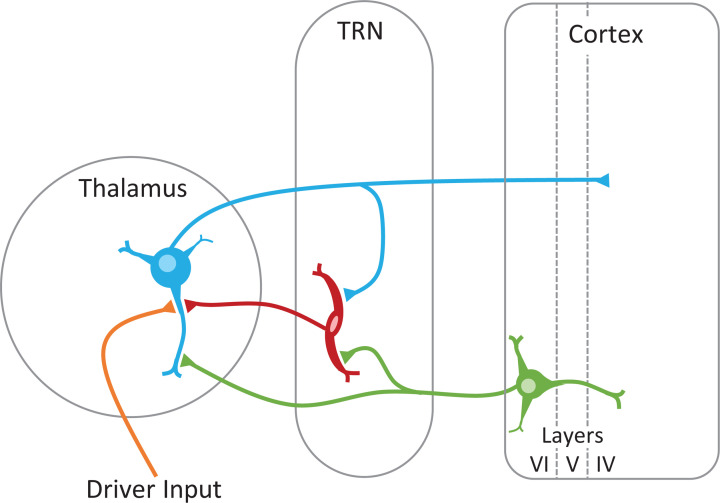
The basic neuronal network common to all thalamic nuclei Each of the major thalamic nuclei in Figure 1B comprise the same basic circuitry. The source of driver inputs dictates the classification of thalamic nuclei as either a first- or higher-order nuclei, with first-order nuclei receiving and processing driver inputs from the periphery, and higher-order nuclei receiving and processing driver inputs from cortical layer V. Driver input – orange; thalamic relay neuron – blue; TRN – red; corticothalamic neuron – green. Abbreviation: TRN, thalamic reticular nucleus.

A basic neuronal network, common to all thalamic nuclei, governs how driver inputs are relayed to the cortex [11–16] (Figure 2). Following innervation by a driver input, thalamic relay neurons send out an excitatory thalamocortical projection to layer IV of the cortex, and in return receive a reciprocal excitatory corticothalamic modulatory projection from cortical layer VI, which modulates how further driver inputs are processed prior to relay [3,5]. These thalamocortical and corticothalamic afferents also innervate the thalamic reticular nucleus (TRN), which in turn sends inhibitory modulatory projections back to the thalamic relay neuron from which it received its innervation (recurrent inhibition) and to surrounding thalamic relay neurons (lateral inhibition) [12,14–16]. The TRN is comprised exclusively of inhibitory neurons [17] and surrounds the entire anteroposterior extent of the dorsal thalamus (Figure 1B).

Investigations of first-order nuclei have enabled the characterisation of this circuitry, as it is possible to physiologically activate sensory driver inputs with exacting fidelity [12,18,19]. This is particularly true for the rodent vibrissal system, which possesses a precise somatotopy with the arrangement of the whiskers of the snout reflected as discrete somatotopic maps in the brainstem as barrellettes, the ventrobasal thalamic nucleus (VB) as barreloids, and the somatosensory cortex as barrels (leading to this cortical region being sometimes referred to as barrel cortex) [20]. Furthermore, the rodent VB is devoid of any additional afferent projections or intrinsic inhibitory interneurons, which can be found in other rodent thalamic nuclei (e.g., the visual lateral geniculate nucleus [LGN]) and most thalamic nuclei of higher mammals such as primates [1,15,21–24]. This high degree of segregation and simplified organisation has therefore made the rodent VB a thalamic nucleus of choice for electrophysiologists to test and understand the basic neuronal network principles underpinning thalamic function.

## An introduction to group II mGlu receptor pharmacology

Glutamate is the major excitatory neurotransmitter in the CNS and acts at both ionotropic glutamate and mGlu receptor types. Ionotropic glutamate receptor activation directly facilitates fast synaptic transmission, whilst the mGlu receptors provide a mechanism for glutamate to fine-tune synaptic activity [25]. Eight mGlu receptor subtypes (mGlu1–8) have been characterised, which have been classified into one of three groups (Groups I–III) dependent upon receptor sequence homology, signal transduction mechanism, and pharmacology [26].

Group II comprises the mGlu2 and mGlu3 receptor subtypes. These Group II mGlu receptors are often found on presynaptic terminals, which when activated ultimately lead to the inhibition of neurotransmitter release from the presynaptic terminal via their coupling to the G_i/o_ intracellular signalling cascade [27]. mGlu receptors native to physiological systems have been found to form obligatory functional dimers that greatly influence the efficiency with which these G-protein signalling cascades are activated [28,29]. These functional dimers take the form of homomeric complexes (i.e., mGlu2–mGlu2 and mGlu3–mGlu3 homodimers), but also heteromeric complexes with each other (i.e., mGlu2–mGlu3 heterodimers), other mGlu receptor subtypes (e.g., mGlu2–mGlu7 heterodimers) and receptors from other G-protein-coupled receptor families (e.g., mGlu2-5HT_2A_ heterodimers) [28–34]. It is important to note however that a number of expression systems have been used to examine mGlu receptor signal transduction and are therefore not necessarily translatable to mGlu receptor signal transduction coupling across different cell types (e.g., neurons vs glia) [35,36].

The Group II mGlu receptor subtypes *de facto* have considerable sequence homology and therefore similar pharmacology [37]. Accordingly, there are a number of commercially available Group II mGlu receptor orthosteric agonists and antagonists, which orthosterically bind to both mGlu2 and mGlu3 receptor subtypes [38], but as of yet few that can differentiate between mGlu2 and mGlu3 receptors. LY2794193 has recently been developed as a selective mGlu3 receptor agonist [39], but has thus far had limited use in the scientific literature [40], and LY395756 is a compound with mixed properties that acts as an antagonist at mGlu2 receptors and an agonist at mGlu3 receptors [41]. This is an unusual pharmacology and as such experiments using LY395756 need careful interpretation [42–46] (note: *N*-acetylaspartateglutamate [NAAG] has been reported to selectively activate mGlu3 receptors [47], although this has not been confirmed in other studies [48] and there are questions regarding the purity of commercially available NAAG preparations [49]). There has been more success in the development of selective compounds, which target allosteric sites of the mGlu2 or mGlu3 receptors, namely positive and negative allosteric modulators (PAMs and NAMs) [50–56]. PAMs act to potentiate the response of a receptor to orthosteric agonists whilst possessing little or no intrinsic agonist activity, and NAMs antagonise the activity of agonists in a noncompetitive fashion by binding to a site distinct from the orthosteric-binding site [25].

The concept of allosteric modulation has received considerable attention in recent years, in part due to the clinical success of the anxiolytic benzodiazepines, which allosterically enhance the function of the ionotropic GABA_A_ receptor [57], but also due to numerous advantages that this compound class has over exogenous orthosteric compounds. Firstly, orthosteric agonists activate their corresponding receptors independently of their physiological state, whereas PAMs act to enhance the action of receptors activated by endogenously released agonist. PAMs therefore increase physiological receptor activation with temporal and spatial relevance, and likely possess a lower side effect profile than orthosteric agonists. For example, the GABA_B_ receptor PAMs CGP7930 and GS39783 [58,59] do not elicit the hypothermic, sedative, and muscle relaxant effects associated with the orthosteric agonist baclofen [60,61]. Secondly, PAMs are thought to induce either no or only low levels of receptor desensitisation, negating the consequence of receptor down-regulation that can occur upon persistent agonist treatment, as has been demonstrated for the GABA_B_ receptor [62]. Finally, many PAMs are highly selective for a specific receptor, given the lack of necessity for allosteric binding sites to be conserved during evolution. This is in contrast with the requirement for the orthosteric site to be conserved for binding of endogenous agonist. However, it is important to consider that this also represents a potential disadvantage for the use of allosteric modulators in experimental design and interpretation. Indeed, although the low evolutionary conservation of allosteric sites facilitates subtype selectivity, it is important to consider that this may result in significant differences across species (e.g., rodents vs humans).

## Group II mGlu receptor locations in thalamic circuitry

**Figure 3:**
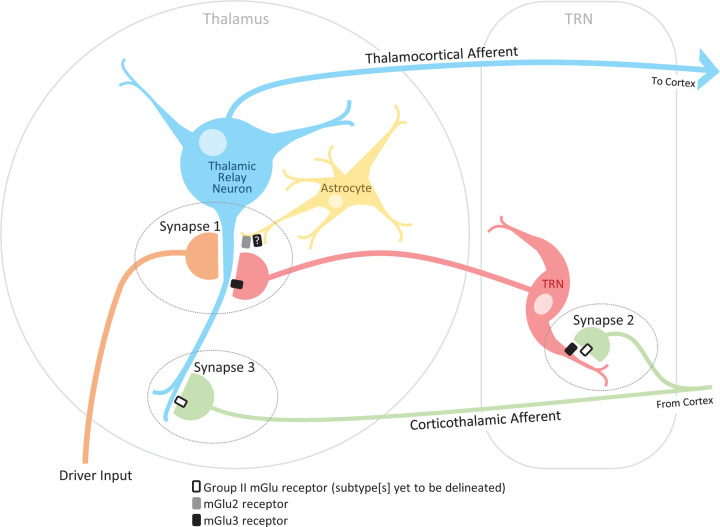
Locations of Group II mGlu receptors within thalamic nuclei Whilst an astrocytic mGlu2 component has been demonstrated at synapse 1 (35), it remains to be evaluated whether there is an accompanying mGlu3 component. At synapses 2 and 3, the identity of the presynaptic Group II mGlu receptors mediating the relevant effects also remains to be delineated. Driver input – orange; thalamic relay neuron – blue; TRN – red; corticothalamic projection – green; astrocyte – yellow.

The mGlu2 receptor has a diffuse protein-staining pattern throughout the thalamus [63] with no significantly increased mRNA signal in any individual thalamic nucleus or the TRN [64]. There is some mGlu2 receptor localisation in cortical layer IV [64]; however, as there is an absence of mGlu2 (and mGlu3) receptor mRNA in thalamic relay neurons [64,65], this is unlikely to be due to presence of mGlu2 receptors on thalamocortical projections. This also indicates a lack of Group II mGlu receptors on thalamocortical projections to the TRN. In contrast, protein staining and mRNA analysis has revealed a highly localised expression of the mGlu3 receptor within the TRN, although little signal in the thalamus itself [64,66,67]. Double-labelling of Group II mGlu receptor protein and the GABAergic marker glutamic acid decarboxylase in the VB of mGlu2 deficient mice found considerable overlap [67], and as the VB lacks inhibitory interneurons [1,15,21–23], this indicates that the mGlu3 receptor is likely present at inhibitory GABAergic TRN terminals. In addition, immunogold particle labelling has also revealed that both mGlu2 and mGlu3 receptors can be found extrasynaptically [64,66,67]. Taken together, these results suggest that mGlu3 receptors are localised primarily on inhibitory TRN terminals that mGlu2 receptors are primarily on glutamatergic cortical terminals (although not thalamocortical in origin), and that both subtypes may be present on glial processes (Figure 3).

## Functions of group II mGlu receptors in thalamic processing

Complementary physiological and pharmacological studies have provided substantial evidence that synaptically activated Group II mGlu receptors are present and functional at key synapses for information processing in the thalamus.

### Synapse 1: the inhibitory modulatory input from the TRN to thalamic relay neurons

*In vivo* electrophysiology studies have demonstrated that Group II mGlu receptor activation with locally applied orthosteric agonists (e.g., 1S,3R-ACPD, L-CCG-I, 2R,4R-APDC) can reduce recurrent inhibition from the TRN to VB thalamic relay neurons upon somatosensory driver stimulation [68–71]. A later *in vitro* study complemented and extended these findings by showing that this Group II mGlu receptor activation with the orthosteric agonist LY354740 reduces inhibitory postsynaptic potential (IPSP) amplitude without affecting the postsynaptic cell membrane properties [72]. This indicates a pre- and/or extrasynaptic localisation of these receptors at the TRN-thalamic relay neuron synapse, which aligns with the findings of the ultrastructural cellular anatomy studies. This inhibitory role has also been shown in the first-order visual thalamic nucleus, the LGN [73], and the higher-order mediodorsal thalamic nucleus [74].

Following development of PAMs for the mGlu2 receptors, an mGlu2 component to this Group II mGlu receptor effect was revealed by local iontophoretic coapplication of the Group II mGlu receptor agonist LY354740 with the mGlu2 PAM LY487379 *in vivo* [75]. As ultrastructural studies indicate a lack of neuronal mGlu2 receptor expression in TRN or thalamic relay neurons, attention turned towards confirming the extrasynaptic localisation of these mGlu2 receptors on surrounding glial processes. *In-vitro* fluorescent calcium imaging in VB slices showed that increases in intracellular astrocytic calcium levels – but not intracellular neuronal calcium levels – could be induced upon application of the Group II mGlu receptor agonist LY354740, which could be further potentiated upon coapplication of the mGlu2 PAM LY487379 [35]. *In vivo*, the glial inhibitor fluorocitrate abolished the mGlu2 PAM LY487379 effect, whilst a considerable neuronal component of the Group II mGlu receptor agonist LY354740 effect was maintained. These data also support the ultrastructural studies (although an astrocytic mGlu3 receptor component remains to be confirmed). However, further *in vitro* and *in vivo* studies have brought into question the relative contribution that these astrocytic mGlu2 receptors make to the reduction in recurrent inhibition from the TRN to the thalamus. *In vitro* experiments in thalamic slices from mGlu3 knockout mice showed a complete ablation of the Group II mGlu receptor agonist LY354740 effect on IPSP amplitude, and use of the mixed mGlu2 receptor agonist/mGlu3 receptor antagonist compound LY395756 *in vivo* demonstrated a majority mGlu3 receptor component to the overall Group II mGlu receptor effect [45]: LY395756 is a more than sevenfold more potent mGlu2 receptor agonist (EC_50_ 0.4 µM) than an mGlu3 receptor antagonist (IC_50_ 2.94 µM) [41], yet the overriding effect of the mixed compound when applied alone was that of antagonism. Indeed, the mGlu2 receptor PAM LY487379 used to reveal the mGlu2 component in earlier studies is itself very effective as it can potentiate a ∼3% of maximal glutamate response (to 1 µM glutamate) up to ∼75% of maximal glutamate response [50] – a 2500% increase meaning that even low levels of mGlu2 receptor activation can be revealed. Therefore, the mGlu2 receptor effect revealed by the potent mGlu2 receptor PAM on reducing recurrent inhibition in the thalamus from the TRN may, under normal physiological conditions, minimally contribute to the overall Group II mGlu receptor effect. Indeed, *in vivo* experiments conducted in the higher-order mediodorsal thalamic nucleus showed that whilst the Group II mGlu receptor agonist LY354740 could reduce inhibition from the TRN upon prefrontal cortex and amygdala driver afferent stimulation, there was no potentiating effect upon coapplication of the mGlu2 receptor PAM LY487379 [74].

In addition to confirming the functional presence of the Group II mGlu receptors at the synapse from the TRN to thalamic relay neurons, these experiments also raise two important concepts. Firstly, they suggest that these Group II mGlu receptors are physiologically activated upon sensory stimulation: the mixed mGlu2 receptor agonist/mGlu3 receptor antagonist compound LY395756 was able to reduce thalamic relay neuron responses from baseline levels, indicating antagonism of endogenous receptor activation [45], and the mGlu2 PAM LY487379 was able to potentiate thalamic relay neuron responses in the absence of locally applied Group II mGlu receptor agonist LY354740 [75], also indicating endogenous receptor activation. The source of this endogenously released glutamate is likely to be from excitatory afferent terminals relaying either driver or modulatory information. Ultrastructural studies indicate that there are no axo-axonic contacts onto inhibitory TRN terminals, and corticothalamic modulatory afferent terminals are not closely associated with TRN terminals in the rat VB [76]. Therefore, transmission from and receptors associated with corticothalamic modulatory afferent terminals are unlikely to be involved in this mechanism. However, ultrastructural studies do indicate that driver afferent terminals are located close to inhibitory TRN afferent terminals and glial processes on the soma or proximal dendrites of thalamic relay neurons [21,76]. Glutamate may therefore be ‘spilling over’ from the driver afferent synapse to activate Group II mGlu receptors localised on glial processes surrounding TRN terminals [77,78] and/or mGlu3 receptors localized on TRN-originating inhibitory axons [67]. This could be leading to a reduction in recurrent inhibition with consequent facilitation of thalamic relay neuron responses to driver afferent stimulation. Similar ‘glutamate spillover’ has been shown to activate Group II mGlu receptors *in vitro* [73,79–82], making it appropriate to suggest that Group II mGlu receptors in the thalamus may be activated via this mechanism *in vivo*.

Secondly, as activation of the astrocytic mGlu2 receptors leads to an increase in intracellular calcium (Ca^2+^) concentration, this suggests mGlu2 receptor coupling to the G_q_, rather than the more usual G_i/o_, G-protein intracellular signal transduction cascade. It is possible that astrocytic mGlu2 receptors are directly coupled to the G_q_ intracellular signal transduction pathway as whilst normally associated with coupling to G_i/o_ G-proteins, there is evidence that mGlu receptors are able to couple to alternative G-proteins in different cell types [83,84]. It is also possible that astrocytic mGlu2 receptors are indirectly coupled to the G_q_ intracellular signal transduction pathway via receptor dimerisation, either with an mGlu receptor, which is traditionally associated with G_q_ intracellular signal transduction (e.g., the Group I mGlu receptors, mGlu1 and mGlu5 [25]), or a different class of G-protein-coupled receptor traditionally associated with G_q_, such as 5HT_2A_ receptors [32–34]. Further research is required to understand the intracellular signal cascade leading to increased intracellular Ca^2+^ concentration evoked upon astrocytic mGlu2 receptor activation.

### Synapse 2: the excitatory input from corticothalamic projections to the TRN

*In vitro* electrophysiology experiments have indicated the presence of Group II mGlu receptors at both the pre- and postsynaptic sites of the corticothalamic-TRN synapse. Group II mGlu receptor agonists (LY379268, S3-C4HPG) were able to elicit hyperpolarisations and increase membrane conduction through potassium leak (K_leak_) channels in TRN neurons [79,85]. This is likely mediated via postsynaptically located mGlu3 receptors as the TRN is devoid of mGlu2 receptor mRNA [64] and the effect persisted in the presence of tetrodotoxin [85]. The Group II mGlu receptor agonist LY379268 was also able to reduce the amplitude of the corticothalamic excitatory postsynaptic current (EPSC), which is likely mediated by presynaptic Group II mGlu receptors as a concurrent enhancement of the paired pulse facilitation ratio – a hallmark of presynaptic activity [86–88] – was observed [79]. The collective effects of this presynaptic inhibition of glutamate release and postsynaptic inhibition of TRN activity will be a disinhibition of thalamic relay neurons as inhibitory input from the TRN will be reduced. Both these pre- and postsynaptic Group II mGlu receptors are thought to be activated under physiologically relevant conditions by glutamate released from corticothalamic presynaptic terminals [79,89].

### Synapse 3: the excitatory modulatory input from corticothalamic projections to thalamic relay neurons

The Group II mGlu receptor agonists LY379268 and DCG-IV were able to decrease corticothalamic EPSC amplitude, which is likely mediated by presynaptic Group II mGlu receptors as high-frequency trains produced a facilitating response that was reduced by the same Group II mGlu receptor agonists and enhanced by the Group II mGlu receptor antagonist LY341495 [73]. These presynaptic receptors are also thought to be activated under physiologically relevant conditions by glutamate released from the corticothalamic presynaptic terminal [73].

It is also worthy to note that Group II mGlu receptors can reduce innervation from inhibitory interneurons in the LGN [90]. There is, however, potentially contrasting evidence to this, which suggests that mGlu receptors can activate the dendritic terminals of inhibitory interneurons of the LGN in the absence of action potentials, thereby inhibiting postsynaptic LGN thalamic relay neurons [91]. However, the mGlu receptor subtypes mediating this effect have not yet been delineated. In addition, the Group II mGlu receptor agonist LY379268 was unable to perturb responses at the sensory driver afferent-thalamic relay neuron synapse in the LGN [73], in accordance with the lack of immunocytochemical evidence for Group II mGlu receptors at this synapse [64,65].

Collectively, the function of the Group II mGlu receptors at these synaptic locations is to reduce the top-down modulation of incoming information from driver afferents in a spatially and temporally specific manner: Group II mGlu receptors at synapses 1 and 2 decrease inhibitory modulation from the TRN, and those at synapse 3 decrease excitatory modulation from the cortex, with innervation from inhibitory interneurons also reduced by Group II mGlu receptor activation in thalamic nuclei, which contain these additional neuronal components.

## Thalamic mGlu2 and mGlu3 receptors in health and disease

The Group II mGlu receptors likely have physiologically relevant functions in the processing of information in the thalamus – functions, which when disrupted could lead to the generation of pathophysiological disease. Here, we discuss the potential roles of thalamic Group II mGlu receptors in states of health and disease.

### Attention

The volume of information entering the brain far exceeds its processing power. Attentional mechanisms are therefore required for the prioritisation of important information. For example, think about the sensory information relayed to the brain in a crowded room – multiple visual and auditory inputs are entering the brain, but it is able to focus attention upon the person (and their voice) that you are talking to. This attentional selection is controlled through both ‘bottom-up’ and ‘top-down’ processes. Bottom-up attentional control is governed by the salience of a stimulus, and top-down modulation by previous experience and expectations [92]. In the thalamus, the synaptic interaction between the driver input, the thalamic relay neuron and the TRN terminal is an example of a bottom-up attentional process to promote sensory detection and discrimination: the firing rate of a thalamic relay neuron receiving a driver input would be relatively enhanced by the attenuation of recurrent – but not lateral – inhibition from the TRN. This selective attenuation can be achieved upon activation of Group II mGlu receptors on TRN terminals and surrounding glial processes (synapse 1) and on pre- and postsynaptic locations at the corticothalamic-TRN synapse (synapse 2). Such a mechanism would enable important information (such as a telephone ringing) to be discerned from background activity (such as a noisy crowd). This mechanism of sensory attention may represent an overarching principle of thalamic function to other first-order and higher-order thalamic nuclei given that mGlu3 receptors mediate the same attenuation of inhibition from the TRN in the higher-order cognitive thalamic nucleus, the mediodorsal thalamus [74].

In addition to bottom-up processes, top-down modulation of driver inputs also occurs in thalamic nuclei via innervation from corticothalamic afferents (synapse 3). Corticothalamic afferents are organised to provide feedback from the cortex that is functionally and spatially aligned with the thalamic relay neurons in order to mirror, and therefore amplify, their activity [93,94]. Such a mechanism would allow higher cortical areas to enhance the discriminative properties of thalamic relay neurons in a way that could increase the salience of responses to novel stimuli. At such synapses, the Group II mGlu receptors would act to preserve the original spatiotemporal quality of the driver input by reducing the mirroring of the corticothalamic innervation and subsequent amplification of thalamic relay neuron excitability.

### Schizophrenia

Schizophrenia affects approximately 1% of the population and is characterized by three symptom profiles: (i) symptoms in addition to normal functioning, termed ‘positive’ symptoms (e.g., hallucinations, delusions); (ii) symptoms that represent an absence of normal functioning, termed ‘negative’ symptoms (e.g., anhedonia, poverty of speech); (iii) symptoms that affect cognitive function (e.g., impaired attention and working memory) [25]. Treatments for schizophrenia have focused on antagonism of the dopamine subtype 2 (D_2_) receptor [95]. However, it is becoming increasingly clear that dysfunction within the dopaminergic system is not sufficient to explain the pathophysiology of the disorder [96]. Indeed, therapies targeting this receptor subtype are often only efficacious in treating the positive symptoms of the disease (and only for a subgroup of patients), whilst exacerbating the negative and cognitive symptoms and inducing a plethora of side effects [95].

In recent years, there has been growing preclinical and clinical evidence for thalamic circuitry disruption in the pathophysiology of schizophrenia, particularly regarding the TRN [97–113]. As the TRN forms neuronal networks with thalamocortical and corticothalamic projections across all thalamic nuclei, TRN dysfunction would result in maladaptations across multiple modalities (e.g., sleep, emotional processing, cognitive performance) that rely on sensory processing and attention, and have been postulated to underlie the generation of hallucinations [100,105,107,114,115]. An emerging correlative theory of the pathophysiology of schizophrenia pertains to maintaining balance between excitatory and inhibitory signals within neuronal circuitries [116–118]. Disruptions of the excitatory–inhibitory balance in thalamic nuclei would have profound effects on how incoming information is processed and subsequently relayed in the thalamus as the usual firing patterns of thalamic relay neurons would be impacted: there are two types of firing patterns displayed by thalamic relay neurons – short-latency tonic responses, which occur at regular rates upon depolarization from resting potential and represent faithful relay of information through the thalamus, and long-latency burst responses that occur at irregular rates in hyperpolarised neurons and do not maintain the original spatiotemporal quality of the driver input [119–123]. Increased inhibition leading to hyperpolarised thalamic relay neurons and subsequent thalamic hypofunction has been associated with schizophrenia disease states in both animal models [124–127] and human imaging studies [128–132]. Reducing inhibition within thalamic nuclei to restore tonic-firing patterns and faithful relay of bottom-up driver inputs may therefore be of therapeutic benefit in the treatment of schizophrenia, such as that which can be achieved by activation of the Group II mGlu receptors to reduce inhibition from the TRN (synapses 1 and 2). Targeting of the mGlu3 receptor subtype specifically is likely of therapeutic importance as it is this Group II mGlu receptor subtype, which appears to majority mediate the effect of thalamic disinhibition in first- and higher-order circuitries [45,74]. Indeed, mGlu3 receptors have been implicated in the aetiological, pathophysiological, and pharmacotherapeutic aspects of schizophrenia [133–135], with polymorphisms in the mGlu3 receptor gene and protein, but not the mGlu2 receptor, detected in patients with schizophrenia [136–138]. mGlu3 PAMs have been identified as compounds of interest in the treatment of schizophrenia [135,139] and are proposed to have enhanced efficacy over direct Group II mGlu receptor agonists, which have had limited success [140–143]. However, no mGlu3 receptor PAMs are yet commercially available, although there are some in development [144].

### Absence epilepsy

Absence epilepsy, which majority affects children [145], is characterised by a regular, bisynchronous, symmetrical, generalised EEG pattern of 2.5–5.5 Hz, which is termed a ‘spike-wave discharge’ [146]. Absence seizures typically manifest as a period of stillness, with the person appearing to stare blankly into space for a short period of time before returning to usual levels of alertness [145]. Whilst the origin of absence seizure activity remains to be determined [147–149], there is strong evidence to suggest thalamic involvement in their generation [150–152], and not just their maintenance and propagation. Current first-line therapies to treat absence epilepsy are typical anticonvulsants [95], which lack specificity due to their modulation of the GABAergic signalling [25]. The Group II mGlu receptors, which have distinct localisations in thalamic circuitry, have therefore been investigated as potential targets for the treatment of absence epilepsy.

The role of the Group II mGlu receptors in animal models of absence epilepsy is however highly debated as the Group II mGlu receptor agonist LY379268 has been found to reduce the duration of spike-wave discharges in a lethargic mouse model [153], but enhance spike-wave discharges in a WAG/Rij rat model [154]. These contrasting findings in two different rodent models of absence epilepsy are difficult to reconcile. Differential expression ratios of Group II mGlu receptor homo- and heterodimers [155] in the corticothalamic network underlying spike-wave discharges in lethargic mice and WAG/Rij rats two models has been cited as a potential explanation [156]. However, recent work with the selective mGlu3 receptor agonist LY2794193 in the WAG/Rij model suggests efficacy in reducing spike-wave discharges [40] in agreement with the lethargic mouse model findings but opposing the previous WAG/Rij findings. Alternatively, or indeed additionally, it is possible that this reflects species differences in Group II mGlu receptor signalling. Clearly, further work with subtype selective compounds is required to fully understand the role that each Group II mGlu receptor subtype plays in modulating spike-wave discharges in absence epilepsy, and it is likely that the effects of a systemically administered mGlu2-selective, mGlu3-selective, or mGlu2/3-pan-selective compound would reflect the relative differential distributions of mGlu2 and/or mGlu3 receptors within the corticothalamic network.

There is also growing evidence for modulation of epileptiform activity in the thalamus by the mGlu7 receptor subtype [157,158]. It is possible that heterodimeric mGlu2–mGlu7 receptor complexes are mediating some of these effects and as such should be considered in future investigations.

### Arthritic pain

Arthritis manifests as swelling and inflammation in a joint, which can become painful. Osteoarthritis occurs when the cartilage lining the ends of bones becomes worn over time, with rheumatoid arthritis a result of an autoimmune disorder where the lining of joints is degraded [159].

mGlu3 receptor expression in the rat TRN can become elevated upon induction of arthritis [160]. As mGlu3 receptor activation can disinhibit thalamic relay neurons, mGlu3 receptor antagonism may be of use in the provision of analgesia in arthritic pain by reducing responsivity to nociceptive driver inputs. Indeed, when applied locally to the TRN, the Group II mGlu receptor antagonist EGLU had an antinociceptive effect in an arthritis pain model [161]. This was likely achieved via antagonism of mGlu3 receptors located: (i) pre- and postsynaptically at the corticothalamic-TRN synapse (synapse 2), which would increase TRN activity and therefore inhibition of somatosensory thalamic relay neurons, and (ii) at the TRN-thalamic relay neuron synapse (synapse 1), which would increase the inhibitory drive from TRN terminals.

## Conclusions

The Group II mGlu receptors are located at key points in thalamic circuitry and likely contribute to the processing of incoming information prior to relay to the cortex. These roles in thalamic function represent potential targets for intervention in disease states including schizophrenia, epilepsy, and pain. Careful consideration is needed during the clinical development of mGlu2 and mGlu3 receptor PAMs to account for heterogeneity between rodent and human mGlu2 and mGlu3 receptor allosteric-binding sites as efficacy may not be translatable across species and act as a potential confounder. Furthermore, as compounds that act as orthosteric antagonists or NAMs at Group II mGlu receptors in other areas of the brain have been identified as potential therapies for other disorders (e.g., depression [82,162]), clinical developers of Group II mGlu receptor compounds need to be aware of the potential for the induction of unwanted side effects.

## Data Availability

Data sharing is not applicable.

## Open Access

Open access for the present article was enabled by the participation of University College London in an all-inclusive *Read & Publish* agreement with Portland Press and the Biochemical Society under a transformative agreement with JISC.

## Abbreviations

CNS: central nervous system
EEG: electroencephalogram
EGLU: (2S)-α-ethylglutamic acid
EPSC: excitatory postsynaptic current
IPSP: inhibitory postsynaptic potential
LGN: lateral geniculate nucleus
mGlu: metabotropic glutamate
NAAG: *N*-acetylaspartateglutamate
NAM: negative allosteric modulator
PAM: positive allosteric modulator
TRN: thalamic reticular nucleus
VB: ventrobasal
